# Anti-drug Antibody Validation Testing and Reporting Harmonization

**DOI:** 10.1208/s12248-021-00649-y

**Published:** 2021-12-01

**Authors:** Heather Myler, João Pedras-Vasconcelos, Kelli Phillips, Charles Scott Hottenstein, Paul Chamberlain, Viswanath Devanaryan, Carol Gleason, Joanne Goodman, Marta Starcevic Manning, Shobha Purushothama, Susan Richards, Honglue Shen, Jad Zoghbi, Lakshmi Amaravadi, Troy Barger, Steven Bowen, Ronald R. Bowsher, Adrienne Clements-Egan, Dong Geng, Theresa J. Goletz, George R. Gunn, William Hallett, Michael E. Hodsdon, Brian M. Janelsins, Vibha Jawa, Szilard Kamondi, Susan Kirshner, Daniel Kramer, Meina Liang, Kathryn Lindley, Susana Liu, ZhenZhen Liu, Jim McNally, Alvydas Mikulskis, Robert Nelson, Mohsen Rajabi Ahbari, Qiang Qu, Jane Ruppel, Veerle Snoeck, An Song, Haoheng Yan, Mark Ware

**Affiliations:** 1 Immunochemistry Department, PPD Laboratories, 2244 Dabney Road, Richmond, Virginia 23230-3323 USA; 2 Product Quality and Immunogenicity, Office of Biotechnology Products, Office of Pharmaceutical Quality, Center for Drugs Evaluation and Research, Food and Drug Administration, Silver Spring, Maryland 20903 USA; 3 Immunogenicity, GlaxoSmithKline Pharmaceuticals, 1250 South Collegeville Road, Collegeville, Pennsylvania 19426 USA; 4 NDA Advisory Services, Ltd., Grove House, Guildford Road, Leatherhead, KT22 9DF Surrey UK; 5 Eisai Inc., 100 Tice Boulevard, Woodcliff Lake, New Jersey 07677 USA; 6 Global Biometric and Data Sciences, Bristol-Myers Squibb, Princeton, New Jersey 08540 USA; 7 Integrated Bioanalysis, Clinical Pharmacology and Safety Sciences, BioPharmaceuticals R&D, AstraZeneca, Cambridge, UK; 8 Bioanalytical Sciences, Amgen Research, Thousand Oaks, California 91320 USA; 9 Diagnostics Accelerator, Alzheimer’s Drug Discovery Foundation, 57W 57th Street, New York, New York USA; 10 Translational Medicine and Early Development, Sanofi, Framingham, Massachusetts 01701 USA; 11 Specialty Bioanalytics, Teva Pharmaceuticals, West Chester, Pennsylvania 19380 USA; 12 Shire Plc- a Takeda Company, Cambridge, Massachusetts 02142 USA; 13 B2S Life Sciences, 97 East Monroe Street, Franklin, Indiana 46131 USA; 14 Janssen BioTherapeutics, Janssen R&D LLC, Spring House, Pennsylvania 19477 USA; 15 Legend Biotech, 10 Knightsbridge Road, Piscataway, New Jersey 08554 USA; 16 Drug Metabolism & Pharmacokinetics, EMD Serono, Billerica, Massachusetts 01821 USA; 17 Laboratory for Experimental Medicine, Eli Lilly and Company, Indianapolis, Indiana 46285 USA; 18 Predictive and Clinical Immunogenicity Pharmacometrics, Pharmacodynamics and Drug Metabolism, Merck and Co., 2000 Galloping Hill Road, Kenilworth, New Jersey 07033 USA; 19 Kamondi Bioanalytical Consultancy, Rheinfelden, Switzerland / Roche Pharma Research & Early Development, Pharmaceutical Sciences, Bioanalytical R&D, Roche Innovation Center, Basel, Switzerland; 20 Translational Medicine and Early Development, Sanofi, Frankfurt am Main, Germany; 21 Integrated Bioanalysis, Clinical Pharmacology and Safety Sciences, BioPharmaceuticals R&D, AstraZeneca, South San Francisco, California USA; 22 BioAgilytix Labs, Durham, North Carolina 27713 USA; 23 Pfizer Inc., 17300 Trans Canada Hwy, Kirkland, Quebec Canada; 24 Clinical Biomarkers, Vertex Pharmaceuticals, Inc., Boston, Massachusetts 02210 USA; 25 Immunochemistry Department, Covance Laboratories Ltd., Harrogate, HG3 1PY UK; 26 Office of Study Integrity and Surveillance, Office of Translational Sciences, Center for Drug Evaluation and Research (CDER), Food and Drug Administration, Silver Spring, Maryland 20993 USA; 27 Global Product Development, Pfizer Inc., Andover, Massachusetts 01810 USA; 28 BioAnalytical Sciences, Genentech, South San Francisco, California USA; 29 Translational Biomarkers and Bioanalysis, UCB Biopharma SRL, B-1420 Braine-l’Alleud, Belgium; 30 Development Sciences, Immune-Onc Therapeutics, Palo Alto, California 94303 USA

**Keywords:** Anti-drug antibodies (ADA), FDA, Immunogenicity, Neutralizing antibodies (NAb), Regulatory guidance, Validation

## Abstract

Evolving immunogenicity assay performance expectations and a lack of harmonized anti-drug antibody validation testing and reporting tools have resulted in significant time spent by health authorities and sponsors on resolving filing queries. Following debate at the American Association of Pharmaceutical Sciences National Biotechnology Conference, a group was formed to address these gaps. Over the last 3 years, 44 members from 29 organizations (including 5 members from Europe and 10 members from FDA) discussed gaps in understanding immunogenicity assay requirements and have developed harmonization tools for use by industry scientists to facilitate filings to health authorities. Herein, this team provides testing and reporting strategies and tools for the following assessments: (1) pre-study validation cut point; (2) in-study cut points, including procedures for applying cut points to mixed populations; (3) system suitability control criteria for in-study plate acceptance; (4) assay sensitivity, including the selection of an appropriate low positive control; (5) specificity, including drug and target tolerance; (6) sample stability that reflects sample storage and handling conditions; (7) assay selectivity to matrix components, including hemolytic, lipemic, and disease state matrices; (8) domain specificity for multi-domain therapeutics; (9) and minimum required dilution and extraction-based sample processing for titer reporting.

**Graphical Abstract:**

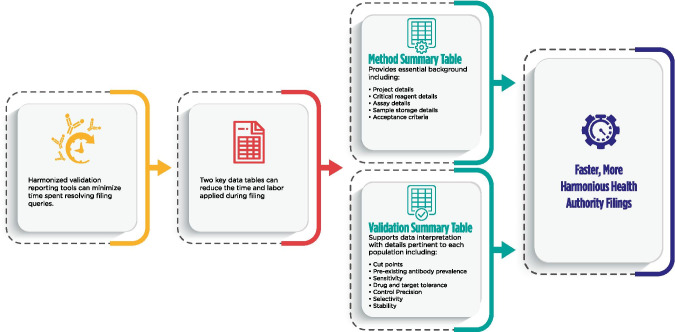

## INTRODUCTION

The purpose of this publication is to provide a model framework for the anti-drug antibody (ADA) validation reports to be included in regulatory submissions and for marketing authorization applications. This model seeks to promote a harmonized scientifically sound approach by presenting illustrative examples that are consistent with current regulatory and pharmacopoeia guidance (1–3) but which does not preclude alternative approaches. This harmonized method report format is intended to facilitate both the sponsor ADA assay validation report generation and the regulatory review process. Neutralizing antibodies are out of the scope of this paper and will be addressed in a separate manuscript.

The proposed model acknowledges that the scope of information to be presented will depend on the therapeutic modality and the immunogenicity risk profile of the modality. While it is not feasible to define acceptance criteria for system suitability controls that reflect the full diversity of appropriate fit-for-purpose practices, we provide examples of approaches that are consistent with current regulatory standards and would suffice for most ADA assay platforms.

It is important to emphasize that many avoidable questions arise during the regulatory review process because pertinent information is either missing or not clearly presented in the method validation report for the ADA assay. For example, drug and target tolerance limits are often reported without discussion of the relevant levels of the interfering factors in the ADA test samples.

## METHOD SUMMARY

A summary of the most relevant method information is included in Table I. The method summary will aid reviewers in understanding the scope of the validations as it pertains to specific projects, methods, validation data including amendments to the initial validation, method use dates, bioanalytical laboratories, and analytes of interest. An outline of critical assay parameters including critical reagent specifications, assay platform, method format, sample pre-treatment including assay minimum required dilution (MRD), confirmatory tier drug concentration, and sample volume required for analysis in each tier along with the sample storage conditions will help reviewers understand the context of the assay. Positive control specifications including source species, type of antibody (monoclonal or polyclonal), and purification details should be included to aid in data interpretation. Control and sample criteria, described in detail below, should be included in this section for each assay tier. Examples of method summary details have been included in italics in Table I for reference. The intent of the Method Summary table is to provide a comprehensive understanding of the method parameters and history, including testing done after the initial pre-study validation, so as to provide regulators with an adequate understanding of the evolution of validation data throughout the life cycle of the assay and the specific evaluations and criteria used for a particular filing. Links to associated reports and any applicable amendments should be included and accessible for reviewers.

**Table I:**
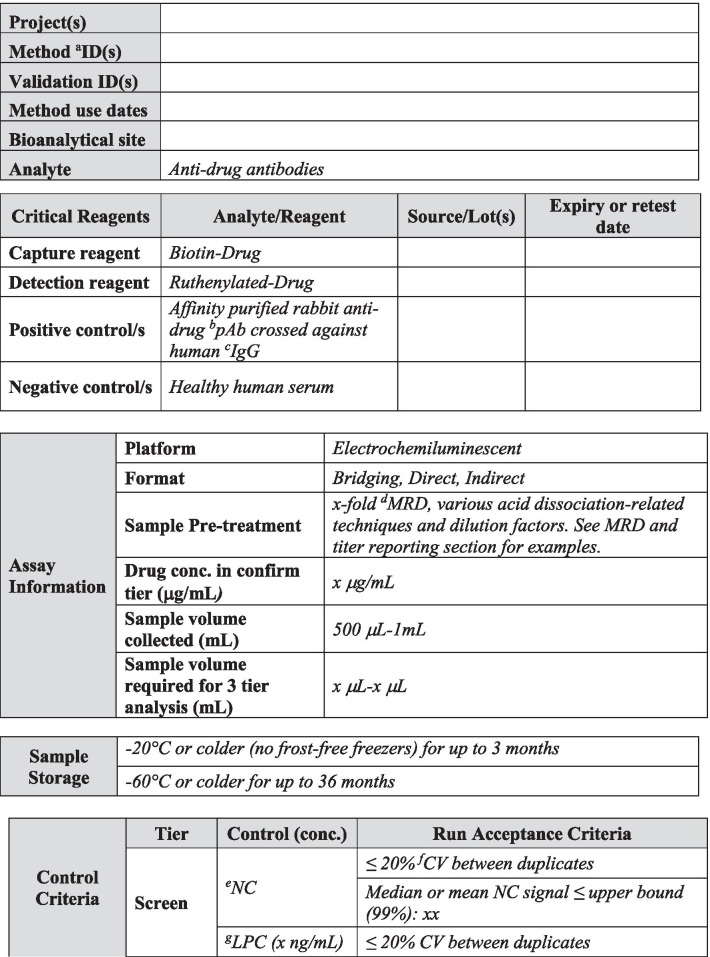

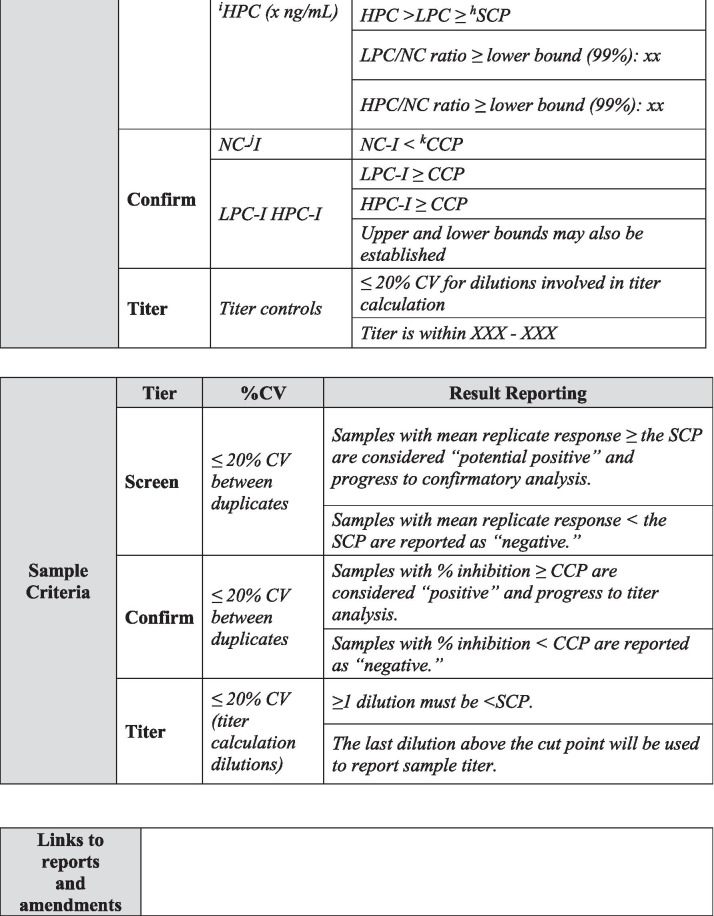
Method Summary

Table I is used to capture salient method details over the life cycle of use. This table is divided into five sections including project details, critical reagent details, assay details, sample storage details, acceptance criteria, and links to reports and associated amendments. The validation report should clearly detail any changes to the method and should be captured in summary in Table I. Critical reagent details should be described in the validation report, including purpose of use, *i.e.*, method development, validation, and/or sample analysis. Pertinent characterization information should be included in the validation report text and in the method summary table. *Text in italics is example text meant to be replaced with actual method detail*

^*a*^*ID indicates identification. *^*b*^*pAb indicates polyclonal antibody. *^*c*^*IgG indicated immunoglobulin. *^*d*^*MRD indicates minimum required dilution. *^*e*^*NC indicates negative control. *^*f*^*CV indicates coefficient of variation. *^*g*^*LPC indicates low positive control. *^*h*^*SCP indicates screening cut point. *^*i*^*HPC indicates high positive control. *^*j*^*I indicates inhibited. *^*k*^*CCP indicates confirmatory cut point*

## SYSTEM SUITABILITY CRITERIA

### Screening Assays

In-study plate acceptance criteria for screening assays that are typically recommended and used in practice during study sample analysis are listed below and described in Table I (4).

1. NC < 99% upper confidence limit for the mean of negative control (NC)
2. LPC/NC > 99% lower confidence limit for the mean of the ratio of low positive control (LPC) to NC
3. HPC/NC > 99% lower confidence limit for the mean of the ratio of high positive control (HPC) to NC
4. HPC/NC > LPC/NC > Screening Cut Point Factor (SCPF)

These criteria can be calculated using data from all the control sample results generated during pre-study validation, prior to the initiation of in-study phase. If sufficient data from control samples were not available to reliably estimate these limits during pre-study validation (< 20 assay plates), additional data from the in-study phase can be utilized to recalculate these limits. In such cases, provisional study phase criteria can be applied until a sufficient dataset is available for a more robust assessment. These limits, along with other assay parameters, also may need to be reassessed during the in-study phase whenever there is a significant change in the assay conditions or reagents.

As described in Shankar *et al*.(4), the 99% upper limit of NC can be calculated as Mean + *t*(0.01,*n* − 1) × SD. In this equation, the mean and standard deviation (SD) are calculated using the data from all the control samples tested during pre-study validation; *t*(0.01,*n* − 1) is the critical value from the 1-sided *t*-distribution with *n* − 1 degrees of freedom corresponding to a 1% error rate, and *n* represents the number of independent replicate NC results used in this evaluation.

The 99% lower limit for the LPC/NC ratio can be calculated as Mean − *t*(0.01,*n* − 1) × SD, where the LPC/NC ratio is calculated by dividing each LPC reportable result by the average of the NC results from the corresponding plate. The mean and SD of these ratios are calculated using data from all the control samples tested during pre-study validation, and *t*(0.01,*n* − 1) and *n* are defined as above. A similar method can be used to calculate the 99% lower limit for the HPC/NC ratios as well. Mean and SD can be evaluated in terms of the appropriately transformed scale (*e.g.*, log), consistent with the transformation used in the screening cut point evaluation of S/N values, and the calculated limits in this transformed scale can be transformed back to the original scale for easier interpretation.

Lower limits for NC and upper limits for the PC/NC ratios are typically not defined because the consequence of “higher than normal” assay signal can result in a higher incidence of reactive samples, which can subsequently prove to be non-specific by the confirmatory assay, and therefore does not impact the ability for the assay to detect false negative samples. When two-sided limits are desired, for example, to monitor assay drift and the separation of LPC and NC or when assay signal is used instead of titer for ADA magnitude determination, the formula provided above can be applied by replacing *t*(0.01,df) with *t*(0.005,df).

Alternate methods for establishing NC and PC control criteria may be suitable, if appropriately justified.

### Confirmation Assays

For confirmatory assays, the plate acceptance criteria can be defined as follows:

1. NC inhibition < 99% upper limit of % inhibition of drug-spiked NC
2. %Inhibition of LPC and HPC > 99% lower limit of % inhibition of drug-spiked LPC and HPC, respectively. Noting that in many cases the inhibition for the HPC is frequently > 95% and a lower limit is based upon a distribution of values in a very small range. Associated justification can be provided if criteria are not set for the HPC.
3. %Inhibition of HPC and LPC > Confirmatory Cut Point.
4. % Inhibition of NC < Confirmatory Cut Point, if the NC matrix is similar to the subject matrix used for the cut point evaluations and devoid of any pre-existing reactivity.

The formula described above for screening assays can be adapted easily for the calculation of these acceptance limits for the confirmatory assays.

### Titration Assays

A key parameter for evaluating the precision of titer assays is the minimum significant ratio, MSR (1). This parameter is estimated using data from a minimum of three assay runs of HPC dilution curves by at least two analysts. The in-study plate acceptance criteria for titration assays can then be defined as follows:

- Titer of HPC in each titration assay plate should be within the MSR of the HPC titer determined during pre-study validation.

For example, if the average HPC titer determined during pre-study validation is 1000, and the corresponding MSR value is 2, then the HPC titer in each titration assay run during the in-study phase should be within twofold of 1000, *i.e.*, within 500 to 2000.

If the MSR value was not calculated during pre-study validation, 2- to threefold difference from the HPC titer can be applied as the in-study acceptance criteria for the titration assays.

## CUT POINTS

The intent of this section is not to provide new guidance on statistical methods for cut point calculations, but to provide clarity on how to present the data so that reviewers and regulatory agencies are able to understand the full context of the validation data. Prior literature on statistical methods has been cited as applicable.

### Pre-study Validation Cut Point Assessments

Today, investigations of unwanted immunogenicity of biotherapeutics are typically performed using a tiered testing approach. Implementation of the industry-standard tiered strategy necessitates determination of specific cut points during pre-study method validation including the tier I screening assay cut point (SCP), the tier 2 confirmatory assay cut point (CCP), the tier 3 titer cut point (TCP), and a domain-specific cut point as applicable to multiple functional domain therapeutics (Table II). Following a risk-based approach, the tier 1 SCP is designed statistically to yield an approximate 5% false positive rate (FPR). This FPR is calculated using baseline samples, after exclusion of samples potentially containing pre-existing antibodies (5, 6). The term “baseline” herein refers to the samples collected prior to the specific therapeutic being dosed for the first time. While this may also be referred to as pre-dose, pre-dose can also be used to describe samples collected prior to each dose in a multiple dose study design. In the tiered testing approach, samples that are classified as potentially positive in tier 1 screening assays will undergo tier 2 confirmation testing. The tier 2 CCP is designed statistically to yield an approximate 1% FPR. Similar to statistical computation of the SCP, tier 2 CCP are determined after exclusion of outlier samples, such as those that contain reactive antibodies. For samples that are confirmed to be positive for reactive antibodies, the tier 3 TCP is used to assign quasi-quantitative titer values. The TCP is set often at a higher level than the SCP (*e.g.*, 1% or 0.1% false positive) to ensure that it is slightly above the lower plateau of the dilution curve allowing for reliable estimation of the titer levels. In the cut point calculation, inclusion of the multiple results for each subject sample (after outlier removal) rather than their average provides an estimate of variance that incorporates intra-sample variability.

**Table II:**
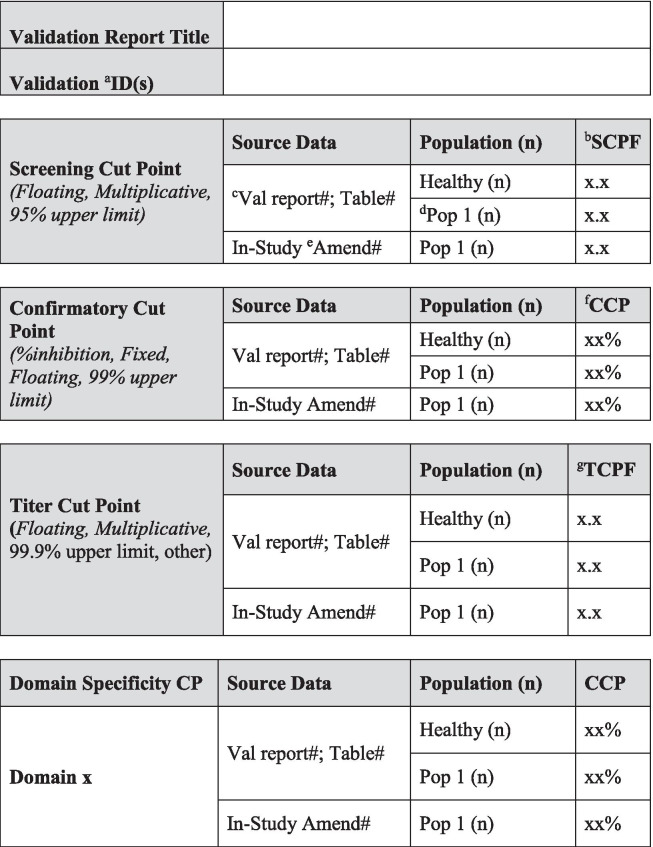

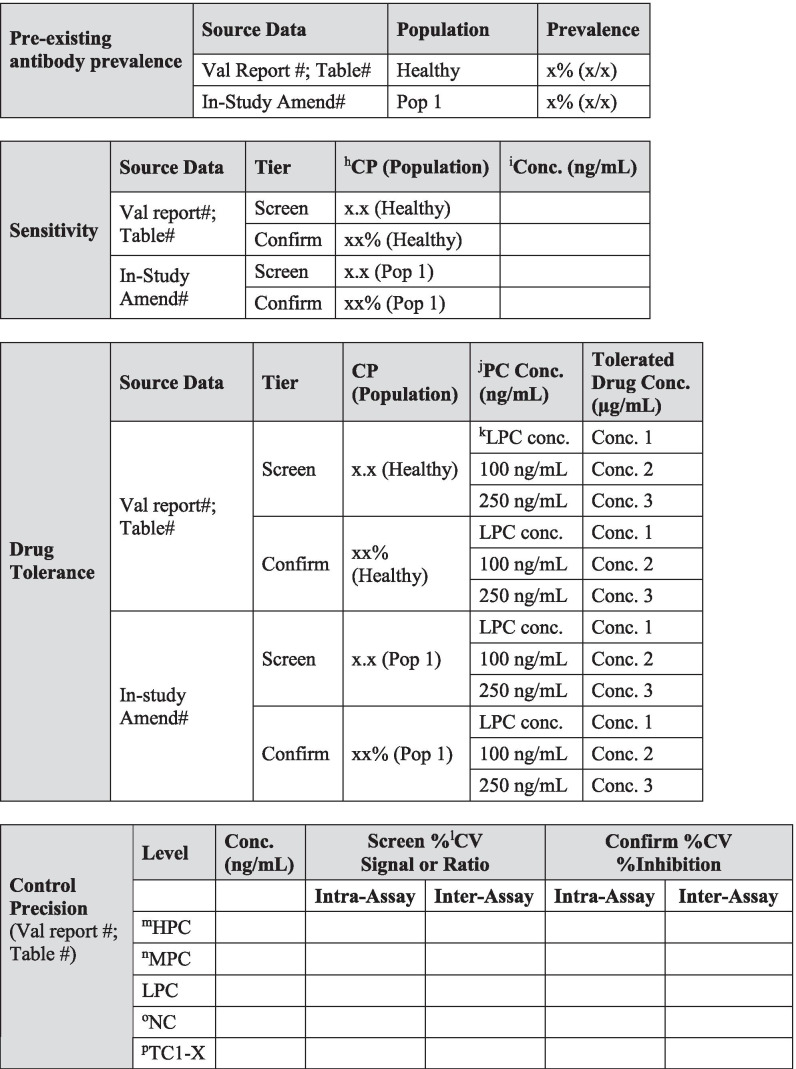

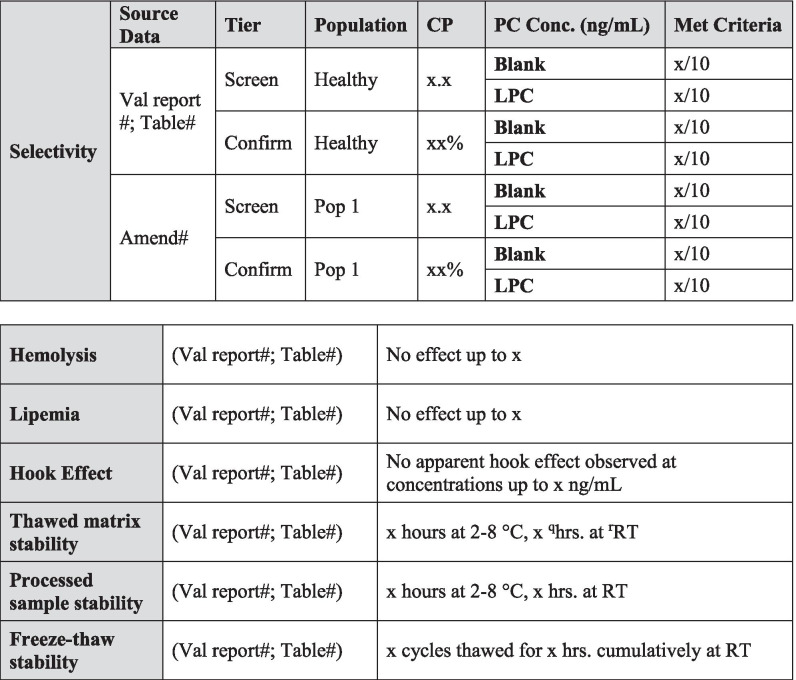
Validation Summary

Table II is used to capture salient validation data details over the life cycle of use. Fields have been included for the first clinical population with the expectation that this table will be updated with details pertinent to further populations and filings. Pop indicates population and should be replaced with the disease state indication. When floating cut point are used, cut point factors should be included in the table for SCP and TCP. For multiple domain biologics, pertinent details for this testing should be added to this table, including domain specificity, sensitivity, and domain-specific CP. Sensitivity, drug tolerance, and target tolerance concentrations should be the concentration in neat matrix. Target tolerance can be reported similarly to that for drug tolerance. Precision is tested and reported across all control levels during validation, including typically 5 titer controls spanning the assay cut point, but only the NC, LPC, and HPC are carried into in sample analysis. Any updates to the control levels as part of assay life cycle management including changes due to in-study cut point application such as increased or decreased LPC, population-specific drug or target tolerance, and population-specific selectivity testing should be clearly noted. Impact assessment should be described for drug tolerance and target tolerance in the validation report pertaining to the levels of drug or target expected in applicable study samples

^*a*^*ID indicates identification. *^*b*^*SCPF indicates screening cut point factor. *^*c*^*Val indicates validation. *^*d*^*Pop indicates population. *^*e*^*Amend indicates amendment. *^*f*^*CCP indicates confirmatory cut point. *^*g*^*TCPF indicates titer cut point factor. *^*h*^*CP indicates cut point. *^*i*^*Conc indicates concentration. *^*j*^*PC indicates positive control. *^*k*^*LPC indicates low positive control. *^*l*^*CV indicates coefficient of variation. *^*m*^*HPC indicates high positive control. *^*n*^*MPC indicates middle positive control. *^*o*^*NC indicates negative control. *^*p*^*TC indicates titer control. *^*q*^*Hrs. indicates hours. *^*r*^*RT indicates room temperature*

Over the past decade, statistical methods for the evaluation of various cut points have been described in a number of publications (1, 4, 5, 7–10). Today, the use of the floating method (4, 5) for SCP calculations by employing a normalization factor and deriving a screening cut point factor by dividing the signal of subject sera by the mean or median NC signal from the same plate has become the common practice to account for the potential drift in the assay results across the assay plates and runs. The benefit of this normalization is readily apparent, if a scatterplot of the average signal of all subjects from each run assay plate *versus* the average signal of the NC samples from the corresponding assay plates shows a positive correlation. A fixed cut point is commonly used for the CCP (4). If the mean % inhibition levels are significantly different between assay runs, a floating approach may also be employed for the CCP as discussed in Devanarayan (5).

While the SCPF can be used for the titer cut point factor (TCPF), we recommend employing a more extreme estimate for the TCP. This is because use of the SCPF can lead to a high degree of variability in the reported titer values. This phenomenon results when the SCP is too low and falls near the lower asymptotic plateau of the dilution curve of the positive control. As described in Devanarayan (5), the TCPF is determined using the same set of data and following the same statistical analysis approach as described for the SCPF evaluation, but with a higher threshold such as the 99.9% threshold (approximate 0.1% FPR) instead of 95% threshold. Confirmed positive samples with signal-to-noise (S/N) values that fall between the SCP and TCP are reported to have a titer equal to the assay’s minimum required dilution (MRD).

A frequent question is whether the cut points determined during pre-study validation frequently derived from commercially available healthy or disease state donors are suitable for use in clinical studies involving clinical subjects and potentially additional disease populations. Separate pre-study validation experiments may not be necessary for each different disease population as the distribution of data (means and variances) may not be significantly different between populations. A simple approach would be to compare the data distributions from pre-study validation experiments *versus* data from at least 20 subjects tested over two or more assay runs from the clinical population (5).

### Pre-study Analysis of Clinical Subpopulations

**Figure 1:**
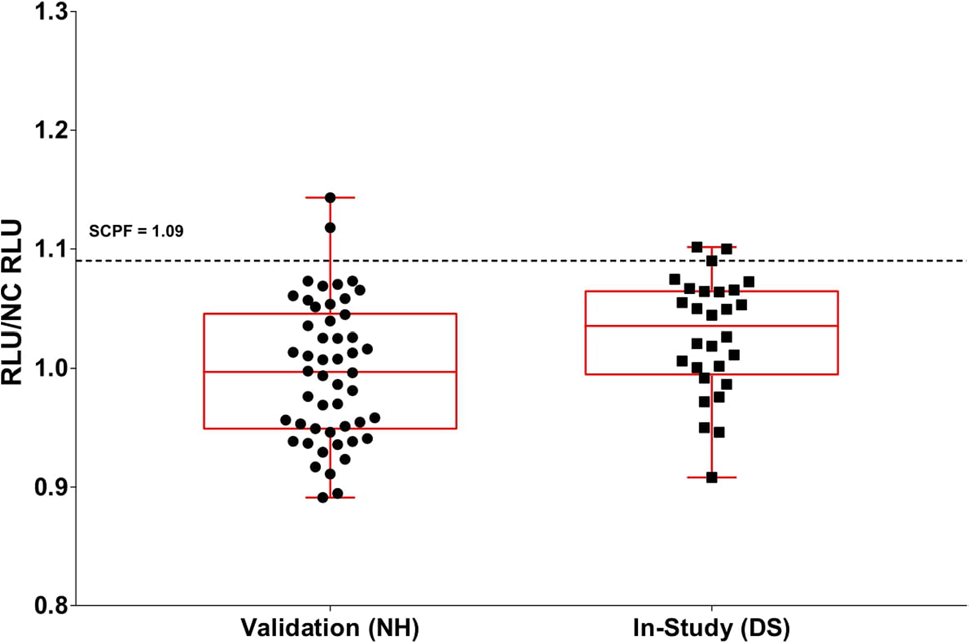
Pre-study validation normal healthy (NH) and in-study disease state (DS) screening sample RLU/NC RLU ratios with the means significantly different and variances not significantly different. Applying the validation screening cut point factor (SCPF) of 1.09 results in the in-study false positive rate of 10%. As this is within a 2 to 11% criteria, the validation SCPF can be used for testing the in-study samples. RLU indicates relative light unit. NC indicates negative control

**Figure 2:**
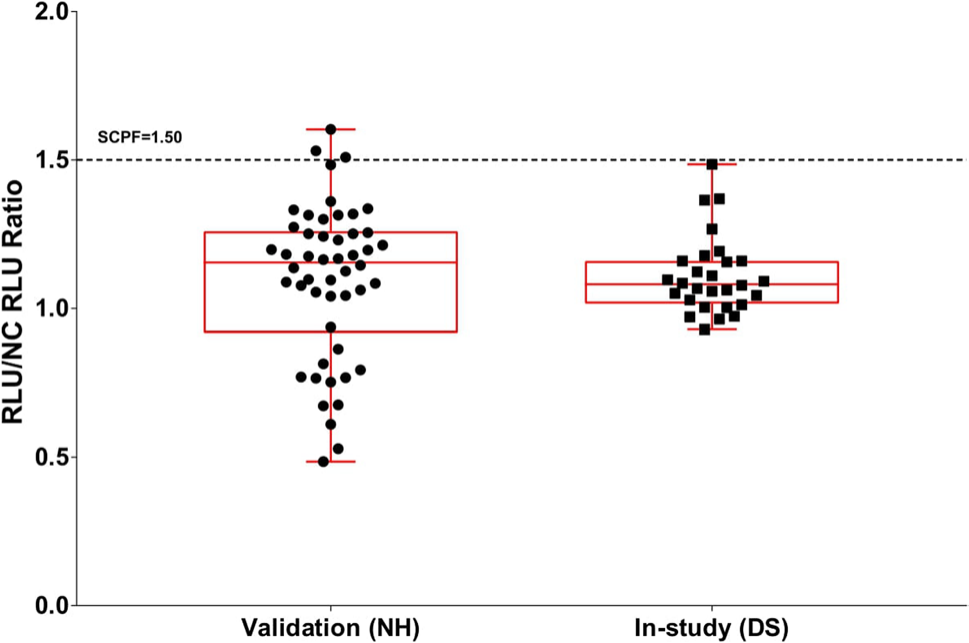
Pre-study validation NH and in-study DS screening sample RLU/NC RLU ratios with means not significantly different and variances significantly different. Applying the validation screening cut point factor (SCPF) of 1.50 results in the in-study false positive rate of 0%, thus requiring the application of an in-study cut point. RLU indicates relative light unit. NC indicates negative control

If different clinical subpopulations (*e.g.*, healthy and disease subjects, or multiple disease subpopulations) are included in the same cut point experiment and they are stratified approximately equally on each assay plate, then the means and variances can be compared to determine whether a common SCP can be applied for all clinical subpopulations or separate SCP are needed. When possible, a minimum of 20–25 samples per patient population is recommended. The means can be compared using an appropriate analysis of variance (ANOVA) model and the variances can be compared via Levene’s test. Visual assessments to compare the distributions (*e.g.*, side-by-side box-plots and superimposed histograms as shown in Figs. 1 and 2) are also recommended to support the statistical comparisons. If the means and variances are not different among the clinical subpopulations, a common SCP can be used for all subpopulations (Fig. 1). If the means or variances are different, then separate SCPs should be determined for each subpopulation (Fig. 2). If the difference in the means or variances among subpopulations are mostly due to one or two subpopulations, then separate SCPs can be calculated for these subpopulations and a common SCP can be applied to the other subpopulations where the means and variances are not different. The same procedure/process described above can be applied for the evaluation of CCPs when multiple clinical subpopulations are included in the same experiment. In-study cut points should be included in Table II for each pertinent patient population along with a reference to the validation amendment and any independent statistical reports.

### Assessment of Screening Assay Cut Point in Samples with Pre-existing Antibodies

Pre-existing reactivity in study subjects’ baseline samples needs to be addressed carefully during pre-study validation when applying the approaches described above for the determination of appropriate SCP. Such samples have higher assay signal response values compared to the majority of drug-naïve ADA-negative samples. If < 20% of baseline samples show evidence of pre-existing antibodies, the usual methods for outlier evaluation and data transformation (5) should be sufficient to bring the distribution of remaining samples close to normality so that the methods for SCP determination can be applied. If, however, pre-existing antibodies are present in more than 20 to 30% of the baseline samples, other approaches may help mitigate the presence of these samples in the cut point calculations. One possible approach is to remove statistical outliers and set the SCP on the remaining dataset. However, this may result in a limited data set for SCP determination and may require further investigation of the root cause of the outliers. A graph of mean percent inhibition from the confirmatory assay *versus* the S/N ratio from the screening assay for each subject sample may help with the assessment of outliers (2, 5). Xue (6) suggested that creating a “pseudo-ADA-negative population” by spiking in enough drug to wipe out the pre-existing antibody signal might be suitable in some cases of high pre-existing antibody observance. The remaining signals of the baseline study samples are then used for SCP assessment. It should be noted however that this approach would preclude the use of a confirmatory assay and CCP using the standard process (4) to assess the specificity of the ADA to study drug. Samples that are identified as reactive in the screening assay would have to proceed directly to the Titer assay.

Pre-existing antibody prevalence should be recorded in Table II for the pre-study validation population used to generate cut points and for each patient population. Any unexpected differences in prevalence can be investigated and/or justified based upon the biological context of the patient population.

### In-Study Cut Points

An in-study cut point may be needed, if the putative positive rate among the pre-dose samples for the patient population being evaluated is outside of the expected false positive rate, which is usually preferred to be within 2–11% range (5, 11, 12), after the exclusion of confirmed positive samples. It should be noted that a reasonable size population is needed to reliably assess the false positive rate. Consultation with a statistician is advised. Additionally, alternative ranges may be set and justified based on the variability and other characteristics of the data on a case by case basis (12). The pre-dose sample results for the current patient population can be compared to those from the pre-study validation (healthy human), or from a previously evaluated disease, using similar methods as described previously for the validation and analysis of clinical subpopulations.

The number of samples required and other design considerations for the evaluation of in-study SCP and CCP depends on several factors, including the number of baseline samples available and adequacy of sample volume for retesting. When possible, a minimum of 50 baseline subject samples representing the diversity of the population is recommended. For larger studies such as those in phase III that tend to be more diverse in terms of demographics and disease characteristics, a minimum of 100 baseline subject samples representing the diversity of the population is recommended. While each subject sample is only tested once, these samples should be distributed and tested across multiple plates and days by at least two analysts. The variability estimate calculated from all sample data will capture both the analytical and biological variation and can therefore be used in the calculation of the cut point. The data assessment should include outlier identification and distribution evaluation prior to the calculation of the cut point from these baseline samples.

Clinical trials in rare disease or pediatric populations may not provide a minimum of 50 baseline samples for SCP evaluation. In such cases, a SCP supplemented with subject samples with similar characteristics, as determined by statistical analysis of available baseline study samples from the clinical population, can be appropriate. When this is not feasible, given the small sample size, all study samples can be tested directly in the confirmatory and titration assays to identify and characterize ADA-positive samples using the CCP and TCP from pre-study validation.

When assessing the need for an in-study SCP and CCP, visual tools such as histograms and boxplots of the two distributions along with a formal statistical assessment of the difference of means (ANOVA) and variances (Levene’s test) would provide further insights on what caused the in-study false positive rate to fall outside the acceptable false positive rate range and provide a supporting tool for the decision to calculate new in-study cut points.

Examples of a couple of different scenarios are illustrated in Figs. 1 and 2. Figure 1 shows side-by-side boxplots for validation normal healthy (NH) sample S/N ratios (*n* = 50) and in-study disease state (DS) baseline study sample S/N ratios (*n* = 28). While the variances are not significantly different, the means are statistically significant (4% difference, *p* < 0.05). However, as the validation SCPF of 1.09 results in only a 10% false positive rate when applied to the in-study baseline samples, separate assessment of the in-study CP is not necessary. Figure 2 illustrates the case where the means are not significantly different, but the variances are significantly different (fourfold different, *p* < 0.05). As the application of validation SCPF of 1.50 on in-study baseline samples results in 0% false positive rate, separate assessment of in-study cut point is necessary.

### In-Study Cut Points for Assessing Multiple Disease States Simultaneously

**Figure 3:**
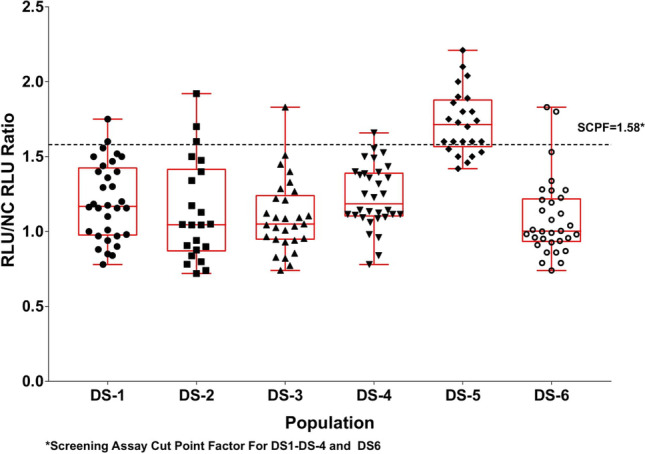
Comparison of in-study baseline samples from multiple disease states (DS) sample RLU/NC RLU ratios. SCPF indicates screening cut point factor. RLU indicates relative light unit. NC indicates negative control

The descriptive statistical and graphical approach can also be used with in-study baseline samples when multiple patient populations are being evaluated simultaneously. To avoid operational variables, baseline samples from each patient population can be run together to prevent confounding any population differences with the plate, analyst, and/or day effects. Figure 3 shows a comparison of baseline values for six oncology populations where sample sizes ranged from 22 to 32 after outlier removal. A common SCP factor for all patient populations is desirable when possible, but this should be justified by evaluating differences in means and variances. For this study, it would be appropriate to use a common SCP for all DS states except possibly DS-5, where the mean and variance are different from those of the other groups and would result in a higher SCP. In this case, the SCP for DS-5 can be estimated using the DS-5 sample mean and standard deviation only. Or, if operationally it is more desirable to have a single cut point across all populations, the most conservative SCP determined with the other four populations could be applied to DS-5.

When working with small sample sizes per DS population (*e.g.*, < 20), the resulting SCP and SCPF should be considered as a starting point, with the cut point(s) re-evaluated when a more suitable sample size for a target population is available.

## SENSITIVITY ASSESSMENT AND SELECTION OF POSITIVE CONTROL CONCENTRATIONS

Assay sensitivity plays a pivotal role when determining if a method is fit-for-purpose. It is commonly defined as the concentration at which the antibody preparation produces an assay readout equal to the assay cut point (13). While reports exist in the literature where ADA responses can be correlated to adverse patient events such as increased infusion reactions and treatment discontinuation (14), adverse patient responses that correlated with ADA concentrations less than 250 ng/mL have been noted in recent years (15, 16). As a result, current FDA guidance has changed the recommended targeted sensitivity for methods used to monitor clinical samples from 250 to 100 ng/mL. The sensitivity should be reported based upon the concentration of antibody in neat matrix and should be reported using mass units in Table II.

Sensitivity should be determined by using ≥ 5 dilutions of a positive control sample that are expected to span the assay cut point in both the screening and confirmatory tiers. For those dilutions spanning the cut point, dilution steps should be small (2- or threefold) to increase the accuracy of interpolation of the ADA concentration equal to the cut point. Since current guidance recommends that the calculated assay sensitivity is used to select the target LPC concentration (2), it is important to capture potential assay variability during runs used to evaluate sensitivity. Therefore, at least six independently prepared sensitivity curves should be evaluated across multiple days by multiple analysts (*n* =  > 6 total). It may even be advantageous to increase the number of curve evaluations to include runs with incubation times at min/max times, multiple instruments, multiple lots of critical reagents, and multiple preparations of non-critical buffers. Data from each run included in the sensitivity assessment are used to calculate the concentration that corresponds to the plate specific screening cut point.

As sensitivity is important to understanding the ability to detect low levels of ADA, the sensitivity section of this manuscript also addresses the impact of the positive control source and selection on determining assay sensitivity, the impact of different disease states and potentially different cut points on sensitivity, the potential for different sensitivities in the screen and confirm tiers, and associated life cycle management concepts. It is very important to note that, ultimately, the LPC and HPC concentrations should cover the lower and upper ends of study sample responses and should be adjusted accordingly using supporting clinical data.

### Positive Control Impact on Sensitivity

Currently, the most prevalent approaches used to calculate assay sensitivity employ a surrogate positive control. This leads to the often-noted observation: sensitivity evaluations may not be representative of assay sensitivity when monitoring an *in vivo* ADA response. While this is true, the use of a surrogate control does not negate the validity of the information gained during sensitivity assessments; it only makes interpreting sensitivity data more complex. When defining assay sensitivity, the positive control source, the matrix source, and assay drug tolerance should be considered. Accordingly, the accessibility of these parameters is imperative when submitting the study report to regulatory authorities (Table II).

Polyclonal antibody (pAb) preparations may have a heterogeneous array of ADA with diverse affinities, avidities, and target epitopes. However, pAb also have their draw backs including potentially non-specific immunoglobulins that co-purify with the specific immunoglobulins, elevating the control concentration and decreasing apparent sensitivity. Additionally, while immunization and antibody purification techniques may select for ADA with higher avidities and less diversity, it is generally accepted that pAb are suitable for use as a positive controls in ADA assays. For human monoclonal antibody (mAb) therapeutics, immunization strategies in non-human species may lead to an overrepresentation of anti-human Fc antibodies, which would not be representative of a human ADA response. Thus, it may be more meaningful to use one or more well-characterized monoclonal anti-idiotype ADA (ideally multiple monoclonal ADA with different affinities to the drug) for characterization of assay parameters or to eliminate pAb that are specific to the hu Fc region during affinity purification.

### In-Study Cut Point Impact on Sensitivity

The calculation of an in-study cut point may be triggered if the false positive rate of baseline (pre-dose) samples does not fall within an acceptable false positive rate range for the assay (*e.g.*, 2–11% excluding confirmed positive samples, as in (5)). This may also indicate that the assay sensitivity reported during pre-study validation, normally using commercially sourced healthy population matrix, may not be representative of the assay sensitivity in disease state matrix and/or clinical subjects. At times, a disease state may trigger the modified regulation and expression of matrix soluble proteins. As a result, matrix collected from clinical subjects may have assay responses that are significantly different than those from the pre-study validation donor population. The evaluation of sensitivity in different populations is recommended when population-specific cut points are required in order to set a relevant LPC to that study population.

### Defining Sensitivity in the Screening Assay

Current guidance also indicates that assessment of assay sensitivity is required for both the screening and confirmation assays. These two assays are expected to demonstrate comparable sensitivity.

For simplicity, it is desirable to use an LPC of the same concentration for both the screening and confirmatory tiers. This is possible if the calculated sensitivity for both tiers is similar, for example, within twofold of one another and ≤ 100 ng/mL. In this case, the LPC would be set at the lowest ADA level that consistently screens and confirms positive. If sensitivity is not similar in both tiers, an appropriate LPC should be prepared for each tier.

### Assay Sensitivity Life Cycle Management

Events that impact sensitivity may occur during the life cycle of a method. These events and any resulting impact should be monitored carefully to ensure the continuity of reported sample results and can be accomplished through control response monitoring. Various factors can attribute to changes in response including the degradation of capture and detection reagents, the introduction of a new NC pool, and the introduction of new capture and detection reagents all of which have the potential to impact assay sensitivity and require adequate bridging and potentially partial validation.

Careful screening techniques during the preparation of a replacement NC pool when the NC pool used in validation is getting close to being depleted can prevent the need to re-assess cut point and assay sensitivity. It is recommended to demonstrate that the performance of replacement NC pool meets the criteria established during the assay validation. If the NC pool used in validation is available and is performing as originally established, the replacement NC pool can be qualified by demonstrating comparability of the responses using multiple replicates of the validation NC pool and replacement NC pool run on the same plate. Or, more comprehensively, comparison of the distribution of results for the replacement NC pool run on several plates to the distribution of results of the validation NC pool across many assays may be done to assure they cover a similar range. To account for any response variance in the NC pool, it is recommended that PCs be prepared in the same pool as the NC.

A new lot of PC will not impact the interpretation of validation results (*i.e.*, the positive control response does not impact unknown sample responses). However, a formal reagent qualification procedure of a new PC lot should be performed to ensure the LPC continues to have the same raw response as the LPC used to set assay acceptance limits. In scenarios when the new lot of PC does not have comparable raw responses, the PC may be titered to match the validated LPC response. Updating the LPC concentration does not indicate that the assay sensitivity has changed but does provide more information about the range of assay sensitivity that may be expected.

All changes to assay parameters as a result of life cycle management should be clearly noted in the validation summary table (Table II).

## DRUG TOLERANCE

The presence of circulating drug in study samples is the most common obstacle to detection of ADA. Drug present in samples competes with ADA assay reagents, results in decreased assay sensitivity, and potentially causes false negative results. Drug interference is especially problematic for mAb therapeutics, which are administered at high doses and have long half-lives. To minimize drug interference, it is important to select sampling time points when drug is at the lowest possible level. Samples should be collected prior to dosing, during the dosing phase at drug trough (immediately prior to the next dose), and at the end of study after an appropriate washout or non-dosing period (approximately five half-lives after the last exposure) (17). Drug tolerance can be improved by optimizing assay conditions such as the MRD, reagent concentrations, and incubation times. In addition, various sample pre-treatment techniques and assay formats, including acid-dissociation (18), solid-phase extraction with acid dissociation (SPEAD) (19), affinity capture elution (ACE) (20), biotin-drug extraction with acid dissociation (BEAD) (21, 22), bead-extraction and heat-dissociation (BEHD) (23), and precipitation and acid-dissociation (PandA) (24), can also be used to overcome drug interference. Disadvantages of these techniques include potential loss of certain types of ADA (25), poor ADA recovery during sample pre-treatment, and worsening of target interference (26). Therefore, the mildest assay conditions that still result in adequate drug tolerance should be chosen. A combination of an appropriate sample collection schedule, sample pre-treatment, and assay optimization can ideally result in the ability of the assay to detect ADA in all true ADA positive study samples, despite the presence of drug.

Unless drug is confirmed to be completely absent in ADA samples, the assessment of drug tolerance is a required component of ADA method validation (2, 3). Validated drug tolerance is highly dependent on the characteristics of the surrogate positive control (4). It is generally understood that the validated drug tolerance may not reflect the actual drug tolerance for all study samples. Of note, it is possible to detect ADA in clinical study samples that have drug levels that exceed the validated drug tolerance limit in cases where very high ADA levels that can overcome drug interference, or if the drug tolerance is overestimated due to the specificity or affinity of the positive control used during validation. However, uncertainty regarding the results may hinder interpretation. Nevertheless, drug tolerance results from validation are useful for understanding the assay’s capabilities and are used by regulatory agencies as part of the evaluation of the suitability of the method for detection of ADA in study samples (2, 3). Other factors, such as risk assessment and clinical consequences of ADA development in patients, may influence regulatory agency expectations regarding drug tolerance. For example, if no clinical consequences are observed in patients with low-level ADA responses, assay drug tolerance may be acceptable even if the expected drug level is tolerated only at ADA concentrations higher than 100 ng/mL. If the desired level of drug tolerance is unachievable after an appropriate effort has been invested into assay optimization, collaborative discussions with regulatory agencies are recommended.

To validate drug tolerance, samples containing positive control (surrogate ADA) and drug are prepared in matrix. Drug concentrations are selected to cover the range of concentrations expected to be present in study samples and should be included in Table II. After drug measurements in study samples become available, additional drug levels may need to be evaluated if observed drug levels are higher than those tested during validation. The selected drug concentrations are spiked into pooled matrix containing positive control at the following recommended concentrations: LPC concentration, 100 ng/mL (FDA’s recommended sensitivity; (2), and at least one additional positive control concentration (*e.g.*, 250 ng/mL). For some unique modalities and/or assay formats, drug present in the study sample may change the background signal and it may be helpful to include a 0 ng/mL sample to demonstrate that the drug does not impact a negative result. Higher positive control ADA concentrations may also be tested if adequate drug tolerance is not demonstrated at these recommended ADA concentrations. Alternatively, a serial dilution of ADA concentrations can be used to determine the assay sensitivity in the presence of drug (1).

Drug tolerance samples should be incubated for at least 1 h to allow the formation of complexes and may be frozen prior to analysis to better represent study sample conditions. Samples are analyzed in both the screening and confirmatory tiers of the ADA assay. One validation run is sufficient if drug tolerance is determined to be in excess of what is required, or if pre-validation data are consistent with validation data. If drug tolerance results are variable or close to method requirements, additional validation runs (*i.e.*, at least 3) may be required to ensure reporting of reliable results. If multiple drug tolerance runs are performed, the median tolerated drug concentration should be reported in the validation summary table (Table II). All data should be included in the drug tolerance data table. The evaluation of drug tolerance in different populations is only recommended when these different populations require the use of population-specific cut points. Drug tolerance does not necessarily need to be re-determined experimentally in matrix from the intended population. Drug tolerance can be calculated by applying the population-specific cut points to existing drug tolerance validation data. In cases when sensitivity curve performance is vastly different in healthy human matrix compared to diseased state matrix, it is advisable to determine drug tolerance in specific populations experimentally.

Final validated drug tolerance results in both assay tiers and for various populations (if applicable) are listed in Table II. Positive control and drug concentrations should be based on mass units in undiluted matrix. The drug tolerance limit can be based conservatively on the highest concentration of drug tested that still produces a positive result in the screening and confirmatory assay at a given positive control concentration. Alternatively, the drug tolerance limit can be interpolated at the screening or confirmatory assay cut point from the drug inhibition curve. If multiple drug tolerance runs are performed, the median tolerated drug concentration should be reported, recognizing that this is an approximation and that the assay drug tolerance may sometimes fall above and below this value. It is recommended that drug tolerance be reported as a spectrum of ADA and drug concentrations and that these results are summarized according to the example in Table II. It is very important that the maximum drug concentrations expected to be present in study samples be described in the text of the validation report and discussed in relation to the validated drug tolerance result.

## TARGET TOLERANCE

Soluble drug targets can be secreted, shed from cell surfaces, or released upon cellular breakdown. The degree of interference due to soluble drug target is related to the assay format, target characteristics (monomer or multimer), disease specific factors, and the concentration of soluble drug target in the sample. Bridging ADA assay formats are particularly prone to target interference, as drug-derived reagents are used to the capture (unlabeled or biotin labeled) and detect the ADA (24, 26–30). Target interference may cause either false negative or false positive results. For example, soluble multimeric targets may mimic the bivalent ADA molecule and cause false positive results even at low concentrations. High background in the screening assay along with a high confirmatory cut point can be an indication of target interference. Different approaches have been used to mitigate target interferences including target removal or blocking with target-specific antibodies/proteins, as well as other sample pretreatment methods (24, 26–32). However, sample manipulation procedures have the potential to introduce new artifacts. Acid dissociation pretreatment, which is often used to improve the drug tolerance of an ADA assay, may worsen target interference by disrupting drug-target complexes and releasing accumulated target (26), or by multimerizing a monomeric target in a sample (24). Sufficiently high levels of monomeric targets also have the ability to saturate drug assay reagents, which lead to potentially false negative results by blocking the detection of ADA that bind at or near the target binding site.

It is imperative to have a good understanding on the target’s biology and the ADA assay in order to interpret the data accurately. Thus, an assessment of target tolerance may be an essential component of an ADA method validation (3). Since the validated target tolerance is highly dependent on the characteristics of the surrogate positive control antibody and the source of soluble target used for validation experiments, it is generally understood that the validated target tolerance may not reflect the actual target tolerance for all study samples. Nevertheless, target tolerance results are useful for understanding assay performance and help interpret testing results. This information can also be quite useful in the interpretation of integrated pharmacokinetic, target, and immunogenicity clinical data (3). Drug treatment may result in target accumulation, which could exacerbate target interference in post-administration samples (32). If this is anticipated, it is important to test anticipated target levels in the presence of anticipated levels of drug at ADA sampling time points.

To validate target tolerance, samples containing various concentrations of soluble target in the absence or presence of positive control ADA (and drug when scientifically justified) are prepared. The soluble target used in the evaluation should be as similar to the native soluble target present in study samples as possible. The preferred source of target may be pooled matrix samples containing known concentrations of endogenous target. However, since actual disease matrix with high endogenous target level is often not available at the time of validation, recombinant soluble target is routinely used as a suitable surrogate in validation experiments. The source of the recombinant target should be selected carefully as the protein may not be representative of the structure of the native soluble target, *e.g.*, some commercially available targets have an Fc fusion, resulting in an unnatural dimer. Concentrations of the target in the validation samples are selected to (1) represent the range of target levels measured in the study population and (2) represent a possible increase in target levels following dosing, due to pharmacological effect or drug-mediated accumulation. To determine the target impact on a positive ADA response, selected target concentrations should be evaluated in the presence of ADA positive control at the following recommended concentrations during validation: 0 ng/mL to evaluate the potential of target cross-reactivity (false positive results), at low positive control concentration and 100 ng/mL (FDA’s recommended sensitivity (2)). Higher ADA concentrations may be tested if adequate target tolerance is not demonstrated at these recommended ADA concentrations.

Target tolerance validation samples should be incubated for at least 1 h to allow the formation of interference complexes and may be frozen prior to analysis to better represent study sample conditions. Target tolerance samples are examined in both the screening and confirmatory tiers of an ADA assay. One validation run is acceptable if target tolerance is determined to be sufficient for future study samples, or if validation data are consistent with pre-validation data. Three validation runs may be required to ensure reliable validation results if target tolerance results are inconclusive. If multiple target tolerance runs are performed, it is suggested that the median tolerated target concentration will be reported in Table II. All data should be included in the target tolerance data tables. The evaluation of target tolerance in different populations is only recommended when these different populations require the use of population-specific cut points. As with drug tolerance, target tolerance does not necessarily need to be re-determined experimentally in matrix from the intended population. It can be approximated by applying the population-specific cut points to existing target tolerance validation data. In cases when sensitivity curve performance is vastly different in healthy human matrix compared to diseased state matrix, it is advisable to determine target tolerance in specific populations experimentally.

The target tolerance limit can be reported as the highest target concentration that does not interfere with antibody detection at a certain positive control concentration or the highest target concentration that does not cross-react in the assay in the absence of ADA and cause a false positive result (4). Alternatively, the tolerated target concentration can also be interpolated at the screening or confirmatory assay cut point from the target concentration curve. Final target tolerance limits in both assay types, various populations (if applicable), and at each control concentration should be included in Table II in a similar manner as drug tolerance results. If target tolerance is demonstrated to be acceptable in the final method, prior to method validation, it may not need to be repeated during method validation. In this case, the results can still be summarized in the validation summary table with the notation that the work was conducted during method development. Associated documentation should be accessible upon request. Positive control and target concentrations should be based on mass units in undiluted matrix.

### Control Precision

Precision of the negative, positive, and titer controls should be evaluated during the method validation and recorded in Table II. Intra-assay precision may be assessed by evaluating independent preparation of positive control levels within a single assay assessment. It is recommended that at least six positive control replicates for each level be included in this assessment. Inter-assay precision may be assessed by using data from all validation runs (*n* =  ≥ 6 replicates per level with multiple analysts in multiple days). For each of these evaluations, it is expected that the response precision, using either raw or normalized values (S/N), be within 20%. Positive control limits, based on precision evaluations, are discussed in the system suitability criteria section.

## SELECTIVITY

It is critical during validation to understand the effect of matrix components within a sample that could potentially interfere with the ability of the assay(s) to correctly assess immunogenicity. Such interferences are evaluated as part of the selectivity assessment. Endogenous and exogenous components may include free hemoglobin (hemolysis), lipids (lipemia), bilirubin, soluble target, rheumatoid factors, Fc receptors, drug, concomitant medications, and pre-existing antibodies (2, 3). It is recognized that sample matrices such as serum or plasma may cause a level of signal alteration in the assay and thus act differently than assay buffer. Therefore, a minimal required dilution is usually employed within the assay to help reduce such effects but may not eliminate them entirely. It is important to consider that samples from disease populations may contain different components from those in healthy matrix and that each disease population may be different. As a result, selectivity testing should be considered when changing into a disease population or between disease populations. Variables such as ethnicity and age are not typically included in selectivity testing but if population differences are observed in study samples, in-study cut point/s and selectivity testing may be warranted.

### Assessment of Selectivity During Pre-study Validation

To determine the selectivity of the assay, varying amounts of positive control antibody are spiked into matrix that potentially contain the interferents of interest, and the ability for the ADA assay to correctly classify selectivity samples as positive or negative is determined. It is recommended that samples from at least ten individuals are used for the selectivity assessment and positive controls are tested at the LPC concentration in both the screening and confirmatory assay tiers. It is acceptable to use the data generated in cut point evaluations to support selectivity assessments. For example, un-spiked samples from cut point runs can be used to determine selectivity at the unspiked (blank) level. Additionally, cut point data can be used to pre-screen for negative samples that can then be used for control-spiked sample selectivity. If positive control-spiked samples are from the same individuals used to perform the cut point assessment, then un-spiked data from that assessment does not need to be repeated in the selectivity experiment. Selectivity is usually evaluated in a single run. Guidance documents from regulatory agencies do not specify acceptance criteria for this validation parameter. It is our recommendation that the LPC-spiked matrix should classify as positive in the screening tier for at least 80% of tested samples and the same 80% or above should also confirm as positive in the confirmatory tier. For the un-spiked samples, at least 80% of samples should classify as negative and NC samples should fulfill acceptance criteria. Selectivity results from all relevant populations should be included in Table II. If pre-existing antibodies are seen in the population, then it is recommended that samples without pre-existing antibodies are utilized for the selectivity assessment. For cases where high levels of pre-existing antibodies are known to be present in the population (*e.g.*, > 20%), a justifiable approach may be to move directly to the titer assay testing tier and traditional selectivity testing may not be a feasible validation parameter. The prevalence of pre-existing antibodies should be included in Table II.

In most cases, the LPC level evaluated in the pre-study validation selectivity assessment will be the same as the level of the assay acceptance LPC that is prepared in pre-screened pooled matrix. However, insufficient performance of this LPC level in selectivity testing may indicate that a higher LPC level is merited for assay acceptance. In such cases, justification for this difference should be included in the validation report.

The FDA recommends assessing selectivity against assay buffer spiked with positive controls. Performing this comparative assessment during method development is considered more suitable. Substantial differences between the buffer and matrix samples may indicate that the assay requires further optimization such as a higher MRD, to be chosen ahead of validation.

### Hemolysis, Bilirubin, and Lipemia

For healthy sample matrices, the presence of hemolysis should be unlikely if the sample is well-prepared and lipemia should be uncommon outside of specific disease states. Ligand binding assays are generally unaffected by hemolysis due to the specific binding of the labeled therapeutic for the ADA and the application of wash steps. However, in some disease populations, the presence of significant levels of hemoglobin, lipids, or bilirubin in study samples may be observed. Healthy matrix can be spiked with whole blood (typically 2–3%), spiked with intralipid or at least 300 mg/dL of triglyceride as an attempt to mimic real samples. For the initial assessment, a single pool may suffice but if the population is likely to have such interferents present then we recommend a minimum of five samples be assessed in one run. As for selectivity in a healthy population, hemolytic, lipemic, etc. matrix should be spiked at LPC concentrations which should screen and confirm positive. Un-spiked matrix and NC should classify as negative. In the case where individuals are used rather than a pool, it is acceptable for 80% of the selectivity samples to meet the required classification. If the assay(s) are shown to be impacted, then assessment and management strategies need to be employed during sample analysis. This may take the form of visual assessment, possibly combined with the use of color charts. Any clearly hemolyzed or lipemic samples should be documented during sample analysis and a priori strategies should be established to determine if (or how) the affected samples are tested. Regulatory authorities may request this information during bioanalytical inspections, and the final bioanalytical study report should include the rates of sample hemolysis or lipemia if rates exceed 10% of samples. Lipid, hemoglobin, and bilirubin levels are frequently evaluated with routine clinical chemistry. However, most labs do not have such testing available and gaining practical access to such information is often logistically challenging.

### Other Interfering Substances

For other interferents such as rheumatoid factor, it can be challenging to determine whether samples contain the interferent as they cannot be assessed by eye. However, there may be opportunity to implement steps at the assay development stage to remove such interferences. This could be the application of techniques such as adding a blocking agent or utilizing a sample pre-treatment step. However, the interference may not be consistent over the course of the clinical study. While it may be possible to add in routine sample pretreatment or assay steps to remove such interferents, the application of these steps routinely to study samples may not be warranted if it is an uncommon observation during assay development in the relevant study population. In this scenario, our recommendation is not to move to routine assessment of such interfering factors during sample validation or in study phase bioanalysis. Nevertheless, if unexpected results are seen during sample analysis, this should trigger an investigation. As described in Mire-Sluis (13), the acceptable degree of interference should be scientifically justified depending on the nature of the samples and significance of the immune response (13).

More recently the subject of biotin interference has become a discussion within the bioanalytical community, driven by an increased uptake of health supplements containing biotin by the general population. It has been shown for some clinical immunoassays utilizing biotin-streptavidin binding interactions that biotin can interfere in the accurate reporting of results (33, 34). While the evidence for impactful interference of biotin in immunogenicity assays at levels seen with these products is yet to be confirmed, this may be a consideration when assessing selectivity and may be evaluated in method development.

### Changing Clinical Population

Moving from one population to another can result in altered assay performance and impact immunogenicity classification. Selectivity may be assessed when changing disease population, especially if other known interferents are likely to be present. In these cases, the cut point may require re-evaluation in the specific population and, if the study-specific cut point is likely to result in significantly different sensitivity, it could be informative to evaluate selectivity, sensitivity, and drug tolerance in the study population, with results documented in Table II. It is recognized that there may be instances where the disease state is rare or limited and not all validation parameters may be feasible to assess. In situations where matrix is truly limited, cut point, drug tolerance, and sensitivity would take priority over the selectivity assessment.

## DOMAIN SPECIFICITY CHARACTERIZATION

### Definition of Multi-domain Biologics

Multi-domain biologics (MDB) are a growing class of therapeutics that typically have a complex mechanism of action with more than one domain, each domain having a specific role or function. Examples of this growing class of therapeutics include Fc fusion proteins, PEGylated proteins, bi-specific antibodies, and antibody drug conjugates. Like other biologics, MDB also elicit a polyclonal immune response with multiple specificities and affinities towards the different domains (35). This in turn can have various degrees of impact on the overall drug activity, pharmacokinetic profile, and safety (35).

### Risk Assessment and the Need for Domain Characterization

The immunogenicity assessment strategy is driven by the molecule’s risk assessment and is a regulatory expectation (2, 3). While out of scope for this paper, it is important to conduct the risk assessment based on patient and product factors keeping in mind some unique considerations for MDB such as the presence of repetitive antigenic structures, presence of neoepitopes or non-natural sequences as a consequence of molecule engineering, the possibility of epitope spreading, and a hapten effect (35, 36). For MDB, the regulatory expectations (2, 3) are clear; a sponsor should consider a determination of immune response to the entire molecule, each of the domains as well as to any neo epitopes to provide a thorough assessment of immunogenicity. Current industry practice is to do this evaluation in the standard tiered fashion in the clinical phases, in particular earlier phases for MDB with limited clinical experience. The risk assessment can be used for determining whether epitope specificity (*i.e.*, which site or peptide sequence) is needed. Depending on the overall assay strategy, the characterization of domain-specific immune responses can also be used when determining the neutralizing potential of antibodies generated against specific domains. Typically, an overall mechanism of action-based determination of neutralizing antibodies (Nab) (*e.g.*, using a single assay) is sufficient unless the risk assessment warrants the need for domain-specific NAb activity. The immunogenicity assessment strategy for MDB can be complex and a variety of assay strategies may be appropriate. Depending on the risk of ADA responses to each domain and the potential biological consequences of such ADA responses, alternative assay strategies, such as a well-designed NAb assay, or a downstream pharmacodynamic assay could provide equivalent or a more meaningful assessments of the impact of ADA responses. Thus, it may be justified to use these assay approaches in place of ADA domain binding ligand binding assays. There is no “one size fits all” strategy and sponsors should pursue the appropriate suite of assays that answer critical questions related to domain-specific ADA.

### Assay Building Blocks — Critical Reagents

Prior to developing assays for assessing the immune responses to MDB, it is important to develop the appropriate critical reagents and an effective reagent life cycle management strategy. Characterizing the domain specificity of an immune response may need additional reagents beyond the general drug reactive positive controls and labeled drug typically associated with non-MDB therapeutics. While not within the scope of this paper, the additional reagents that may need to be generated are domain-specific positive controls, domain-specific competitor molecules with adequate characterization, and domain-specific capture/detection reagents. As reagent generation and characterization are time consuming and resource intensive, our recommendation is to determine the ADA bioanalytical strategy and generate these specialized reagents early in clinical development.

### Conducting the Domain Specificity Assessment

A common approach for domain specificity assessment of ADA often begins with the tiered approach of screen, confirm, and titer of ADA responses against the whole drug product. After confirmation of positive ADA to the entire drug, domain characterization is often done via spiking of domain-specific competitor molecules in the confirmatory tier of the ADA assay to identify the immunodominant regions of the drug. The % inhibition is evaluated and if the % inhibition obtained is > the confirmatory cut point, the sample is considered positive for ADA against the domain of interest. For MDB, it is important to assess whether the confirmatory cut point for the domain is different than that for the entire drug. This is frequently observed, leading to multiple confirmatory cut points with the entire MDB molecule used to generate the first tier of confirmatory cut points, followed by spiking with each domain-specific competitor to determine the domain-specific cut points (35). Each cut point should be recorded in Table II. Via this approach, only qualitative results can be obtained, *i.e.*, positive *versus* negative for the investigated domain.

The advantage of this method is that one assay needs to be developed/validated. However, this approach might be prone to producing false negative signals for domain specificity when a polyclonal response to multiple domains is present (35). The exact degree of signal reduction in the domain specificity confirmation set-up is impossible to translate to the amount of ADA present to a specific domain. Unlike the confirmatory set-up with the entire MDB molecule, the % inhibition obtained after spiking with a specific domain is not a reflection of the amount of ADA towards that specific domain present but reflects the proportion of ADA towards the domain of interest in the total polyclonal ADA mixture. As a consequence, when ADA specific to multiple domains co-exist and are present in different proportions in a sample, domain-specific ADA types present in lower proportion might be left undetected, resulting in false negative reported results for domain specificity as competition with the domain reagent will produce an insufficiently strong reduction in the assay signal (35). This may be partially mitigated by using specific confirmatory cut points for each domain.

Careful evaluation of the risk of false negative results needs to be considered and independent screening and confirmatory assays should be developed if detection of a minor fraction of the polyclonal response to a particular domain of the MDB is required (36). While this ensures that the entirety of the immune response is captured, it requires the development and validation of multiple assays which may not add benefit to clinical impact assessment.

### Hook Effect/Linearity of Response

The maximum concentration of ADA that may be detected should also be evaluated during method validation. For a quantitative type assays, hook effect and dilutional linearity samples that have response results that correlate to sample concentration can help to establish the assay range. In contrast, the hook effects detected in ADA screening and confirmatory assays are usually only problematic if they cause the sample to be inappropriately classified as ADA negative. In this scenario, samples may have to be diluted into the assay range for appropriate confirmatory and titer determination. For ADA assessments, a hook effect or lack of linearity of response can usually be detected through sufficient titration during study phase bioanalysis where the titer level should appropriately reflect the ADA response. While validation testing using the surrogate PC to understand hook effect is informative, the PC cannot represent the diversity of the study samples, and titration data can lead to further understanding and applicable scientifically justified mitigating approaches. If adjustments to the method are needed to accommodate hook affects, this detail should be included in the bioanalytical plan, described in the validation report and noted in Table II.

## SAMPLE STABILITY

To ensure sample integrity over the timeframe of bioanalysis, it is critical to maintain appropriate chain of custody, storage, and handling of clinical study samples. Inadequate sample handling along this path may impact the ability to appropriately detect ADA in the sample as well as potentially resulting in titer changes. Sample processing and shipment should be controlled and conducted using defined procedures. As blood samples are generally processed into serum or plasma and stored frozen for later bioanalysis, freezer temperatures must be continuously monitored to ensure proper storage conditions for samples (generally − 20 °C or − 80 °C). The freezers should be maintained according to the manufacturer’s recommendations, and have a preventive maintenance program in place. Multiple sources, including published literature, the Clinical and Laboratory Standards Institute (CLSI), and International Society for Biological and Environmental Repositories (ISBER), provide information regarding practices for sample management (37).

The purpose of sample stability assessments during ADA assay validation is to validate the process of handling study samples to ensure their integrity. The recommendations for this evaluation are based on the anticipated conditions of sample handling and storage during the bioanalysis phase in the testing laboratory. As it may not be practical to evaluate stability on a subset of clinical study samples, positive controls prepared in the appropriate matrix provide a reasonable approach for the intended purpose. It is recognized that the PC serve as a surrogate for the actual clinical study samples. To generate samples for validation stability studies, affinity-purified antibodies developed from hyper-immunized animals or monoclonal antibodies should be spiked into human serum or plasma matrix to represent surrogate study samples at the low and high levels of positive control. In addition, use of ADA titer stability control samples should be considered and may be informative when performing longitudinal analysis in long-term studies. A sufficient number of aliquots should be prepared to allow the use of independent aliquots for each sample when analyzed in replicate (generally duplicates) and at each respective time point or assessment. A NC sample could be included but is considered optional as it is not expected to change after exposure to stress conditions. If available, prior to registrational studies, a pool of ADA-positive patient sera from earlier studies may also be used for stability assessments but this is not required. In this case, the study informed-consent form should incorporate language allowing the use of clinical study samples as reagents in the assays for which they were intended (*i.e.*, immunogenicity); pooling and de-identification are recommended.

ADA stability assessments should include the evaluation of short-term stability (both bench-top and 2 to 8 °C) and freeze–thaw (F/T) stability. Long-term stability is not required as several published studies from vaccine research, as well as internal industry studies, document that immunoglobulins are stable for several years when maintained under controlled storage conditions (38, 39). Additional reports from diagnostic immunology further support general stability of antibodies (40–43).

Short-term stability has two components, bench-top and 2 to 8 °C. The aim for bench-top stability is to demonstrate that samples are stable when left at room temperature beyond the duration of expected sample preparation time. It is generally recommended that maintaining samples up to 24 h at room temperature should be evaluated. To perform the study, up to three independent aliquots of the HPC and LPC are thawed at room temperature and maintained on the bench for up to 24 h at which time a new aliquot is thawed (time 0) and the bioanalysis performed. The aim for 2 to 8 °C stability is to assess samples stored under refrigerated conditions once thawed. The assessment and duration depend on the laboratory processes. Samples should be stable up to 24 h at 2 to 8 °C or up to 72 h if laboratory practices allow holding samples for an extended period, *e.g.*, over a weekend. Independent aliquots of the stressed HPC and LPC should be compared to freshly thawed samples, time 0.

The F/T stability study provides information concerning how many F/T cycles the same sample aliquot may undergo and still be acceptable for bioanalysis. Generally, up to three independent aliquots of the HPC and LPC will be repeatedly frozen and thawed for five or more cycles. The initial freeze should be for at least 24 h and all subsequent freezes for at least 12 h, with each thaw step for at least 1 h at room temperature. Generally, all samples will be analyzed at one time. An alternative practice is to analyze time 0 (freshly thawed) and only the last F/T cycle, depending on assay reproducibility. In this case, if the last F/T cycle sample fails, then the intermediate F/T cycle samples can be analyzed.

The acceptance criteria used for sample stability can vary but it is important that these criteria be defined in advance of performing the studies. Several approaches are currently in use. They include as follows: (a) stability samples produce the expected result (positive or negative for ADA) and (b) recovery of stressed sample based on the assay signal is within 80–120% compared to the assay signal for the time 0 sample. When using the later approach, it is also advised to evaluate any time-related trends that may indicate lack of sample integrity. When using three independent aliquots, the mean response of these aliquots needs to be within the validated acceptance range of the respective controls.

## MRD AND TITER REPORTING

As discussed earlier, the presence of matrix components can impact the detection of ADA. Sample processing, such as MRD and dissociation techniques, can be used to improve specificity and reduce such matrix effects. This processing should be factored into the calculations of titers when reporting titers (1–4, 7).

With the development of new and more complex biotherapeutic modalities, immunogenicity assays have evolved beyond the typical bridging immunoassay. Complex immunogenicity assay methods have been developed and implemented to achieve acceptable assay sensitivity, improved drug tolerance, or reduced target interference (18–24). Many of the more complicated methods incorporate extraction, precipitation, or purification steps as part of the sample treatment process and determining the total sample dilution might not be as straightforward as simpler assay formats.

Currently, there is no clear guidance on how to calculate MRD or equivalent in validation documents and submissions causing inconsistent practices within the industry. Incorporation of all sample dilutions, inclusion of only the initial dilution, or no consideration for any sample dilution are all observed across the industry. In an attempt to best comply with regulatory expectations, the ADA harmonization team recommends the inclusion of all sample dilutions in the calculation of titer as recommended by regulators (2). Examples are provided herein as representations of some of the more common methods and can serve as a guidance for how to perform these calculations.

In principle, the MRD should correspond to the amount of an individual sample neat matrix in the overall dilution. All dilutions through the final capture step must be accounted for regardless of whether a sample is diluted in simple buffers, matrix, or reagent containing diluent. For example, in a traditional bridging immunoassay where a sample is initially diluted 1/10 (in buffer or acid) followed by a 1/5 dilution with the conjugate mixture addition, the MRD is considered 1/50 when accounting for all dilutions made. The minimum sample titer is also considered to be 1/50.

More complicated methodologies should account for all steps such as extraction, precipitation, and concentration, through the addition of the processed sample to the final incubation plate or surface for detection. For the purposes of comprehensive dilution calculation, full recovery at each step is assumed when performing these more complicated methodologies.

The published PandA method (24) describes an immune complex formation step where 10 µL of sample is mixed with 40 µL of buffer containing excess drug (1/5 initial sample dilution) followed by a precipitation agent addition at a 1/1 ratio in the same plate (cumulative dilution of 1/10). Following a series of washes and spin downs, the acid dissociation and pellet reconstitution are done with 250 µL of reagent prior to capturing on the final surface for detection (cumulative dilution of 1/25). In this example, the cumulative dilution and minimum titer reported are established as 1/25 and any additional sample dilutions performed for determining the antibody titer (pre- or post-treatment) should be additionally included when reporting titer. For example, if samples are further titered 1/2 serially following the last step (prior to coating on the plate), the titer scheme will be as follows (1/25, 1/50, 1/100, etc.). In the PandA example, all sample dilutions and treatments are being performed in the same plate allowing for an easier calculation of the cumulative dilution. An initial sample volume of 10 µL of sample reconstituted with 250 µL acid at the last step before plating corresponds to a total dilution of 1/25 (Table III).

**Table III:** PandA Titer Calculation (*PandA Indicates Precipitation and Acid-Dissociation*)

Step	Description	Step dilution	Cumulative dilution
Complex formation	*10 µL of sample* + *40 µL of drug*	*1/5*	*1/5*
Precipitation	*Add 50 µL of PEG*^*b*^* to above*	*1/2*	*1/10*
Acid dissociation	*After washes, add 250 µL of acid to reconstitute pellet and dissociate complexes*	*1/2.5*	*1/25*
	*Coat samples on high bind plate and remaining steps*	*No further dilution*	*1/25*

For samples diluted further, add the applicable fold dilution for titer reporting, for example, 1/25, 1/50, and 1/100, if 1/2 scheme is followed. ^*b*^*PEG indicates polyethylene glycol.* Source: PandA (Zoghbi, *J. J Immunol Methods*. 2015 Nov; 426:62–9)

The SPEAD method (19) however includes various steps where samples are transferred from plate to plate during the treatment steps (Table IV). In such methods, the calculation of the cumulative dilution should be done systematically. In the published procedure (19), an initial sample dilution of 1/4 in buffer followed by addition of a biotin labeled drug at a 1/1 ratio or a 1/2 further dilution, corresponding to a 1/8 cumulative dilution at this stage. At the subsequent step, 100 µL of this mixture is added to a streptavidin plate and incubated. After incubation and a series of washes, 60 µL of acid is added to dissociate. At this step, the sample is concentrated back at a ratio of 1.67-fold resulting in a cumulative dilution back to 1/4.8. The subsequent transfer of 50 µL of sample to another plate with 150 µL of a neutralizing reagent results in a fourfold dilution (cumulative dilution of 1/19.2). That sample preparation is then coated on the final surface for detection. In this example, the MRD and minimum titer reported are established as 1/19.2 and any additional sample dilution performed for determining the antibody titer (pre- or post-treatment) should be factored into that value. Although not necessary, a minor adjustment to one of the volumes of a particular step can result in a whole number if desired. For example, changing the volume of acid from 60 to 62.5 µL will result in a cumulative dilution of 1/5 at that step and a subsequent overall MRD of 1/20. Regardless of the practice of adjusting the volume or not to allow for whole number MRD values, the actual titer value should be reported.

**Table IV:** SPEAD Titer Calculation (*SPEAD Indicates Solid-Phase Extraction with Acid Dissociation*)

	Step	Description	Step dilution	Cumulative dilution
Day 1	Sample predilution	*1/4 dilution of sample*	*1/4*	*1/4*
	Sample pretreatment 1	*Biotin-drug complex: equal volume of sample and biotin-drug*	*1/2*	*1/8*
Day 2	Sample pretreatment 2	*Streptavidin: biotin-drug complex capture (100 µL of prior step)*	*No dilution*	*1/8*
		*Acid dissociation (60 µL of acid (2/1); concentrated back to 1/4; 50 µL transferred)*	*1.67/1*	*1/4.8*
		*Sample neutralization: 150 µL of base added to 50 µL of acid from prior step*	*1/4*	*1/19.2*
	Sample analysis	*Coat neutralized sample, block, enzyme-HRP drug, substrate*	*No dilution*	*1/19.2*

For samples diluted further, add the applicable fold dilution for titer reporting, for example, 1/19.2, 1/38.4, and 1/76.8, if 1/2 scheme is followed

## DISCUSSION

The most important distinguishing feature of ADA assays is their quasi-quantitative nature as there is no authentic calibration reference standard representative of ADAs developed in patient population. Although surrogate positive control reagents are used to estimate relative sensitivity, their specificity and affinity/avidity may not be relevant to the analyte being measured and clinical relevance may only be established retrospectively by analyzing the relationship of clinical sample ADA test results to clinical end-points.

Despite these limitations, it is possible to assess suitability of an ADA assay if sufficient data on sources of bias are clearly presented in the method validation report. Understanding the influence of the selected MRD on signal-to-noise ratio in test matrix from relevant clinical populations, and biases associated with target and drug tolerance and other interfering factors, enables detectability of a defined surrogate positive control to be assessed in a manner that models the way in which the assay is to be applied. Definition of valid assay cut-points for each respective tier of the testing scheme then enables objective identification of treatment-emergent signals. Demonstration that the assay can distinguish ADA that is reactive with individual moieties of a multi-domain protein or a drug conjugate supports validity to detect treatment-related responses to the different parts of the biotherapeutic.

To be complete, the method validation report should represent a self-standing document that contains all of the information that is required to assess suitability for the proposed applications — incorporating method performance characteristics for all relevant clinical populations, considering potential differences in levels of interfering factors, including the drug itself. It can be helpful to provide a brief summary of critical findings (*e.g.*, signal-to-noise ratio at different MRDs) from the method development phase in the introductory section of the method validation report. Rationale for choice of sample pre-treatment steps and critical reagents including source/method of preparation of the surrogate positive control antibody may also be explained to assist regulators. And listing of batch numbers for all critical reagents is essential. Arguably, a tabular summary that presents information in a consistent format can be the most valuable point of reference for the regulatory reviewer.

## CONCLUSIONS

The purpose of this article is to improve consistency, clarity, and completeness of information presented in the method validation report for ADA assays by building on experience gained to date by industry and regulators. The recommendations are intended to facilitate the processes of preparation and review of the method validation report by providing model templates in conjunction with practical advice on populating the data fields.

Table I presents a quick reference summary of the methodological details, while Table II defines the requisite data fields to be completed for each relevant assay performance criterion. These formats have been designed to meet current regulatory standards and industry practice. Data field definitions are specific to diverse molecular types, including multi-domain therapeutic proteins such as antibody–drug conjugates. Therefore, some data fields may not be relevant for a particular product type. The tabular formats are intended to be updated to reflect the evolution of the method during clinical development and the post-authorization life cycle.

## Declarations

### Disclaimer

The views expressed in this article are those of the authors and do not reflect official policy of the US FDA. No official endorsement by the FDA is intended or should be inferred.
